# Phosphorous extraction and heavy metal separation from sewage sludge ash by two-compartment electrodialysis in an upscaled tube reactor

**DOI:** 10.1007/s11356-023-30384-0

**Published:** 2023-10-24

**Authors:** Lisbeth M. Ottosen, Dines Thornberg

**Affiliations:** 1 Department of Environmental and Resource Engineering, DTU Sustain, Building 118, 2800 Lyngby, Denmark; 2 Biofos A/S, Refshalevej 250, 1432 Copenhagen, Denmark

**Keywords:** Sewage sludge ash, Phosphorous, Electrodialysis, Heavy metals, Extraction, Recovery

## Abstract

Two-compartment electrodialytic extraction (2C-ED) is a one-step process for the simultaneous phosphorous extraction and separation of heavy metals from sewage sludge ash (SSA). The process is driven by an applied electric DC field, which can be supplied from renewable sources. The proof-of-concept of the method was conducted in small laboratory cells; however, upscaling to a continuous 2C-ED process, which additionally can treat SSA suspensions at a low liquid-to-solid (L:S) ratio, requires a new design. This paper presents such a new design. In principle, ED consists of two compartments separated by a cation exchange membrane. One compartment contains a suspension of SSA in water and the anode. A cathode is placed in the other compartment. Electrolysis at the anode acidifies the suspension causing the dissolution of phosphorous and heavy metals. The heavy metals are separated from the suspension by electromigration into the catholyte, whereas the dissolved phosphorous remains in the dispersion solution. In the new design, the SSA was suspended in a tube-shaped reactor with the cation exchange membrane covering the outside. The reactor was placed in a container with the catholyte. Periodically turning off the reactor kept SSA in suspension even at a low L:S ratio without corners and pockets where the SSA otherwise tends to settle. Five 2C-ED experiments were conducted with 1.5 to 3 kg SSA at varying currents and durations. Up to 89% P was extracted. The extracted P was concentrated in the dispersion solution of the SSA suspension, where the obtained P-related concentrations of heavy metals were far below the limiting values for spreading on agricultural land. The experiments underlined that treating the SSA in a suspension with a low L:S ratio is advantageous. A comparison to previous laboratory experiments in small cells treating 50 g SSA shows a significantly more efficient use of the applied current in the new reactor setup. Thus, the new reactor design for 2C-ED fulfilled the set criteria for the operation and did additionally result in a higher efficiency than the laboratory setups, i.e., the design can be the first step towards an upscaling.

## Introduction

Phosphorous (P) is in the EU list of critical raw materials based on two indicators: high supply risk and equally high economic importance (European Commission 2020). P is essential and irreplaceable to all life. Most P-fertilizers originate from mining phosphate rock deposits—a finite and non-renewable resource. With an expanding global population relying on decreasing and deteriorating P resources, developing technologies for improved P recovery is becoming an increasingly urgent environmental, economic, and societal issue (Melia et al. 2017). The rising cost of P rock extraction will inevitably favor the development of such technologies (Melia et al. 2017).

Sewage sludge incineration is practiced in Europe, North America, and Asia (Ma and Rosen 2021), and sewage sludge ash (SSA) is developing into an increasingly important secondary resource for P recovery. Sewage sludge incineration is extensively practiced in European countries such as the Netherlands, Switzerland, Austria, and Germany (Herzel et al. 2016), where legislative developments prohibit the direct spreading of sewage sludge on agricultural land and opt for mandatory P recovery from the SSA. In Denmark, the government’s resource strategy already set a target in 2013 for 80% P recovery from sewage sludge by 2018 (Regeringen 2013). This 80% target sets the optimization criteria for P recovery from SSA in the present work.

For the recovery, wet extraction in acid where the P is leached in H_2_SO_4_, HNO_3_, H_3_PO_4_, or organic acids has been studied extensively (Gorazda et al. 2017). A challenge of acid extraction is the simultaneous extraction of heavy metals and (Ottosen et al. 2013; Herzel et al. 2016). The European Green Deal stimulates the recycling of finite resources but simultaneously underlines a zero-pollution ambition. Consequently, the contamination levels of recovered nutrients from SSA must be kept at a minimum. Technologies of Cu, Ni, Pb, and Zn separation from ashes or liquid phosphoric extract have to be investigated to allow a broad implementation of a P recovery process (Cieślik and Konieczka 2017). Different methods for the separation of P and heavy metals after acid extraction have been suggested, e.g., pH adjustment (Takahashi et al. 2001; Franz 2008), sulfide precipitation (Franz 2008), cation exchange (Franz 2008; Donatello et al. 2010), electrodialysis (Fang et al. 2023), and membrane technologies with new membrane types developed for the purpose (Paltrinieri et al. 2019; Khadem Modarresi et al. 2021).

Another option than acid extraction and subsequent separation is the one-step two-compartment electrodialytic extraction (2C-ED) from the SSA, which enables simultaneous P extraction and heavy metal separation. The 2C-ED process is based on an applied electric DC field over a suspension of SSA in water. Supplying the current directly from photovoltaics or wind may be an option. Hereby, renewable energy is the main supply to run the process. Previously, it was shown that more than 95% P was extracted from two different SSAs by 2C-ED, and the heavy metal content relative to P content in the filtrate by far meets the limiting values for the use of industrial wastes as fertilizers (Ottosen et al. 2016). Li et al. (2018) pointed out the potential added value in utilizing the residual SSA after P extraction in construction materials. Suspending the SSA in H_2_SO_4_ (Ebbers et al. 2015) or hydrolyzed polymaleic anhydride (Sun et al. 2022) during ED may hamper the utilization of the SSA. In line with utilizing the SSA after P recovery, it was shown that the residual SSA after 2C-ED has the potential for use as cement replacement in concrete (Kappel et al. 2018) or clay replacement in bricks (Ottosen et al. 2020). Following these encouraging results on P extraction, heavy metal separation, and production of raw material for use in construction materials, an upscaling of the 2C-ED method is initiated. This paper reports the first step.

### Principle of 2C-ED

**Fig. 1:**
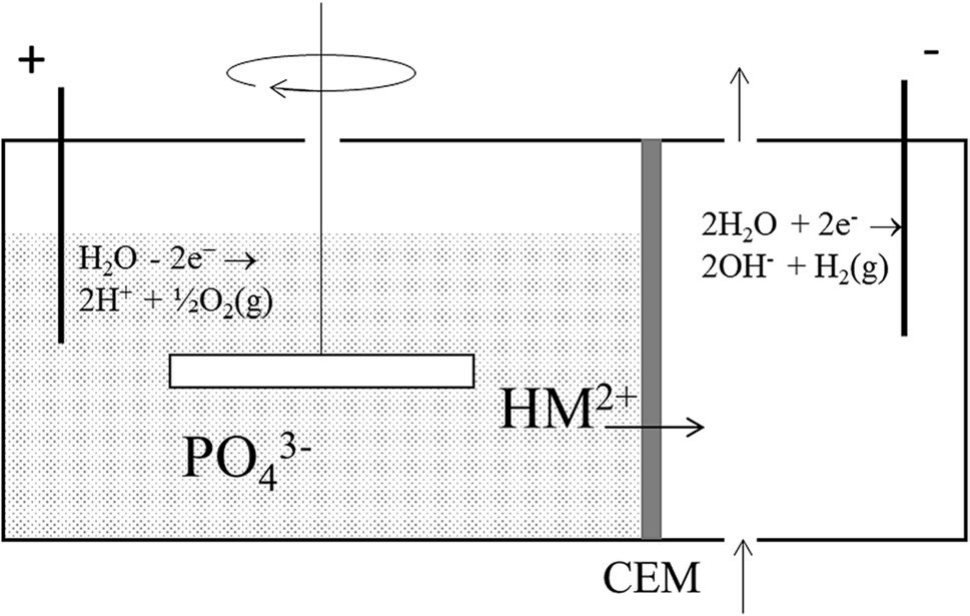
Laboratory cell for 2C-ED. The anode is placed in the compartment with the SSA solution which is separated from the compartment with the cathode by a cation exchange membrane (CEM) (based on figure in (Ottosen et al. 2015))

The 2C-ED laboratory cell was developed for the simultaneous extraction of P and separation of heavy metals from SSA. The steps leading to the 2C-ED cell are described in (Ottosen et al. 2016), and the method was patented (Ottosen et al. 2015). The laboratory cells used in the previous works consist of two compartments, each with an inert electrode (Fig. 1). A cation exchange membrane (CEM) separates the two compartments. The SSA is suspended in water in the compartment with the anode, and the SSA is kept suspended during the extraction by an overhead stirrer. The cathode is placed in the other compartment in an electrolyte solution, which is circulated between the compartment and a flask.

When the electric DC field is applied, the electrical conductance is converted to ionic conductance at the electrode/electrolyte interface through the electrode reactions; H_2_O → 2H^+^  + ½O_2_ (g) + 2e^−^ at the anode and 2H_2_O + 2e^−^  → 2OH^−^  + H_2_ (g) at the cathode. Thus, the SSA suspension will gradually acidify due to the electrolysis at the anode. The acidification results in heavy metals and P dissolution from the SSA. The positively charged heavy metal ions are transported by electromigration over the CEM and concentrate in the cathode compartment, whereas the extracted P remains dissolved in the dispersion solution as negatively or uncharged compounds (H_3_PO_4_, H_2_PO_4_^−^, HPO_4_^2−^, or PO_4_^3−^ dependent on the prevailing pH). With this, simultaneous P extraction and heavy metal separation are obtained (Ottosen et al. 2016).

The extraction due to the acidification by electrolysis does not selectively target P and heavy metals, which means that other chemical elements also are extracted during 2C-ED. About 50% of the SSA is dissolved by the end of the treatment. Next to P, the major chemical elements in SSAs per mass are Ca, Al, Fe, and Si (Kappel et al. 2018). Calcium phosphates are generally reported in SSAs, and they were shown to dissolve during 2C-ED (Kappel et al. 2018). The extracted Ca electromigrates as cations into the catholyte and less than 4% remains in the dispersion solution at the end of the process (Ottosen et al. 2023). Part of the Al from the SSA also dissolves generally about 70% at the end of the treatment, of which about 20–25% remains in the dispersion solution (Ottosen et al. 2023). As Al can precipitate or form charged compounds with the extracted P, Al can potentially influence the extraction result. The target of 80% P in the dispersion solution has been obtained (Ottosen et al. 2016, 2023); however, the slight loss of P into the catholyte (less than 10% (Ottosen et al. 2016, 2023)) might be linked to positively charged P-Al complexes as discussed in Ottosen et al. (2014). Quartz and hematite are the dominant Si- and Fe-containing minerals reported in SSAs, and these two minerals are the major minerals left in the SSA after the 2C-ED process (Kappel et al. 2018). XRF analysis showed that the concentrations of Fe_2_O_3_ and SiO_2_ increased in the SSA during 2C-ED (Fe from 15.7 to 27.3% and Si from 18.6 to 39.4%) (Kappel et al. 2018) revealing that the Fe- or Si-containing particles are largely not dissolved, which is in consistency reported in Ottosen et al. (2014). On the contrary, the CaO content was reported to decrease from 20.1 to 1.0% (Kappel et al. 2018). The relations between the P concentration and Ca, Al, and Fe concentrations in the dispersion solution at the end of the 2C-ED treatment were 0.22, 0.08, and 0.01, respectively (Ottosen et al. 2016). Thus, the P concentration is by far the highest in the dispersion solution compared to the other major elements, which is an advantage in further processing into fertilizer. The general dissolution of the SSA, however, means that part of the applied current is consumed in the side effect of dissolving part of the SSA, which does not contain P or heavy metals. Still, these other elements are not hindering the essential extraction and separation processes in 2C-ED.

### Considerations towards upscaling

The major potential drop in the 2C-ED laboratory cell was found to be over the CEM, and significantly influencing this potential drop is the gradient of potential, conductivity, and pH (Ottosen and Lima 2021). Increasing the CEM area relative to the volume of SSA suspension is a way to decrease the potential drop over a unit area of the CEM (when keeping the process variables the same). During the 2C-ED, the pH and conductivity gradients over the CEM level out as the suspension reaches acidic levels. Here, however, the protons will be important current carriers, which is a waste of energy for the overall recovery process. Previous work has emphasized that the highest energy efficiency for P recovery for the 2C-ED process itself can be obtained if thicker suspensions are treated (low liquid to solid (L:S) ratio) (Ottosen and Lima 2021; Ottosen et al. 2023). The development of 2C-ED has so far been carried out based on findings obtained in small laboratory cells treating 25–50 g of SSA (L:S ratio of 14 to 7). In the laboratory cells, the SSA is kept suspended by an overhead stirrer, and in these cells, settling of the SSA occurs when experiments are conducted at a lower L:S ratio than 7. Treating an SSA suspension with a lower L:S ratio also benefits the P concentration in the dispersion solution being higher after treatment and the volume of the dispersion solution is less per unit SSA treated. When suspended, the SSA settles fast and into a compact layer due to the high particle density, large fraction in the size of fine sand, and irregular shapes (general characteristics for SSAs (Dhir et al. 2017)). This means that keeping the SSA particles in suspension during 2C-ED needs attention. The 2C-ED process is inefficient without stirring (Ottosen et al. 2017). Still, periodical stirring is sufficient, and extraction and separation results comparable to experiments with continuous stirring were obtained by stirring 4 times a day for 1 h (Ottosen et al. 2017). Periodical stirring is advantageous to lower energy consumption compared to continuous stirring.

A single experiment with an upscale of the laboratory cell for 2C-ED to a setup treating 3 kg SSA (at L:S 10.3) was made to produce enough residual SSA for investigating the possible use as cement replacement (Kappel et al. 2018). Two cathode units formed as cassettes were placed at the sides of a box with the SSA suspension and anode in between. To keep the SSA suspended, three overhead stirrers were used. The results were similar to those obtained in the small laboratory cells (about 90% P extraction) (Kappel et al. 2018); however, the SSA tended to settle in the corners, and from time to time, manual interference was needed to get this SSA into suspension again. An upscale should not contain areas without movement where the SSA suspension tends to settle, especially not following the requirement to treat low L:S ratio suspensions.

Previously, a pilot-scale plant for three-compartment electrodialytic (3C-ED) stabilization of heavy metals in municipal solid waste incineration air pollution control (MSWI-APC) residues was designed and tested (Kirkelund et al. 2010; Jensen et al. 2015). In 3C-ED, both electrodes are placed in separate compartments, and the anode and cathode compartments are separated from the ash suspension with anion or cation exchange membranes, respectively. In the 3C-ED pilot plant, the ash suspension (L:S ratio of 10) was pumped through a membrane stack with spacers of 5 mm, and the basic idea was to have a high membrane area relative to the suspension volume (Kirkelund et al. 2010; Jensen et al. 2015). Due to the overall differences in 2C-ED and 3C-ED, the developed pilot plant cannot be used for SSA, but there are experiences obtained to consider. The SSA particles are much coarser than MSWI-APC residues, and to treat suspension with a low L:S ratio, it is foreseen that clogging would be a major issue if using a similar system with pumping SSA through thin spacers (or compartments). However, the idea of the 3C-ED plant to offer a continuous process (Kirkelund et al. 2010; Jensen et al. 2015) is advantageous to the operation of a full-scale plant.

### Aim of present work

The current work aims to design and test a new setup for 2C-ED. The new design is the next step towards upscaling the process, and it shall accommodate the previous relevant findings from the small laboratory cells and previous experiences from upscaling of 2C-ED treatment of ashes. Summing up experiences from previous work, the up-scaled 2C-ED setup shall (1) enable treatment of SSA suspensions with a low L:S ratio, (2) be without corners and pockets where the SSA will tend to settle, (3) enable periodic stirring, (4) the membrane area per unit SSA suspension should be kept high, and (5) the design should support further upscaling to a continuous process.

## Materials and methods

### The new design

**Fig. 2:**
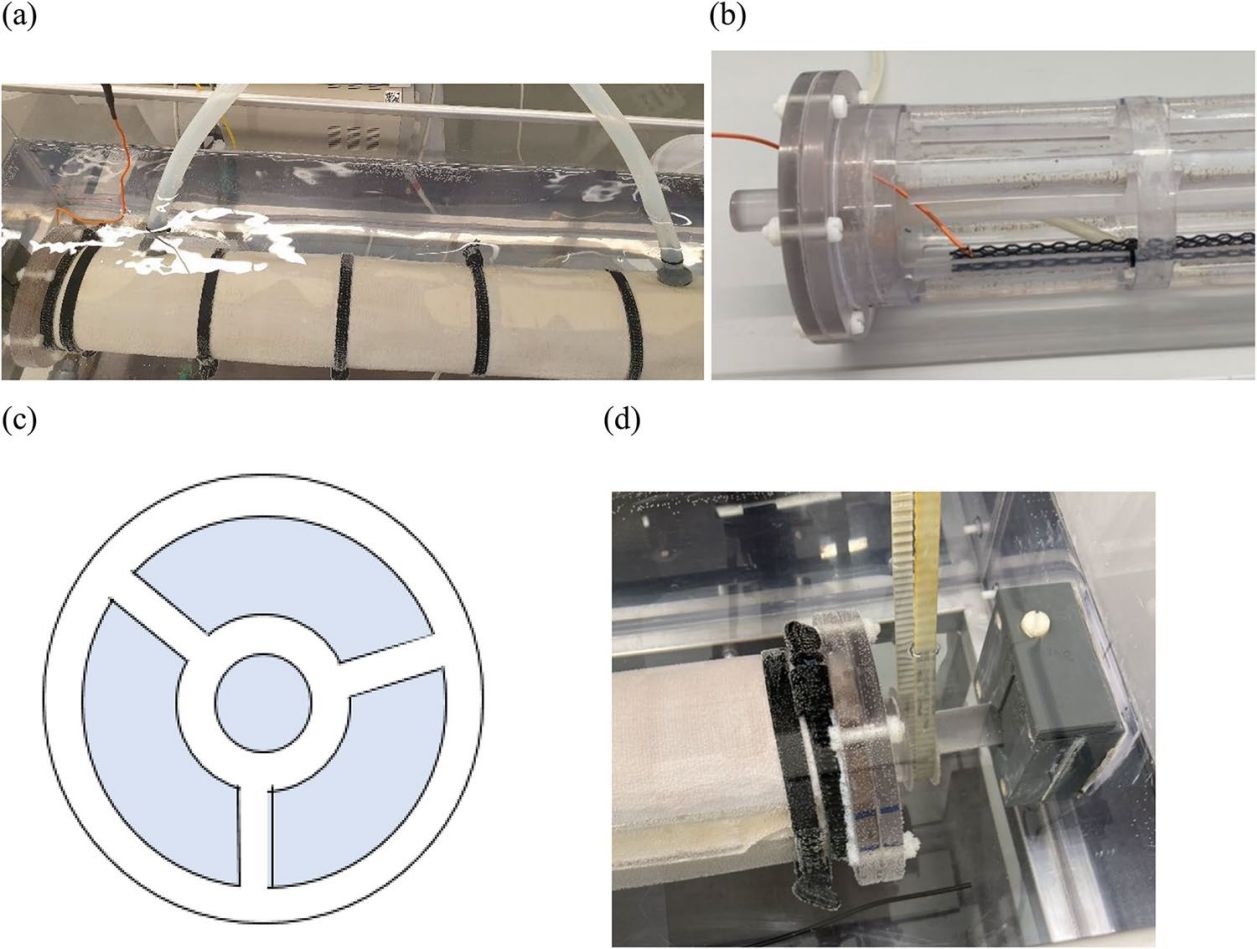
**a** The ED reactor with the SSA suspension placed in the container with the cathode. **b** The internal tube with flange, lid, anode mesh, and holes in the side of the tube, **c** internal support in the center of the tube, where the rod for stabilization and the turns pass through the center, and **d** the timing belt was connecting the motor and reactor

The new design for the 2C-ED meeting the requirements was based on a rotating reactor placed in a container (see Fig. 2a). The suspended SSA was the internal part of the reactor, whereas the catholyte was the solution in which the reactor was placed, i.e., the anode was inside the reactor, and the cathode was in the solution outside the reactor. The anode was a mixed metal oxide (MMO) ribbon mesh, and it was placed close to the bottom of the tube. The outer surface of the reactor was a cation exchange membrane, which was held in place by a supporting, perforated Plexiglas tube (Fig. 2b) and plastic bands on the outside (Fig. 2a). The internal support tube has 2-cm baffles, bent 90° into the tube, hindering the ash from settling on the tube. The reactor was connected to a motor by a timing belt (Fig. 2d). The motor was programmed to allow a periodic turn forth and back of the tube, shifting left and right at 160° each way, ensuring that the anode was in the suspension at all times of the experiment, even if the tube was not filled with suspension.

This design fulfills the requirements set in paragraph 1.3. The rotating tube with small internal baffles allowed ED treatment of a suspension with a low L:S ratio and periodic movement without corners where the SSA can settle. The tube design (long and narrow tube) gives a higher membrane area relative to the volume of the ash suspension compared to treating the same volume of suspension in a shorter reactor with a larger diameter. The reactor design can be developed into a continuous process in that several longer tubes can be placed in the same reactor, and the SSA suspension can be fed into one end and emptied in the other. The transport of SSA through the tubes can be obtained by placing them at an angle compared to horizontally.

### Details on 2C-ED reactor

The reactor was 93 cm long with an outer diameter of 12 cm. It was made entirely of Plexiglas. The thickness of the tube was 0.4 cm, i.e., the internal diameter was 11.2 cm. Six holes (12.5 cm long and 5 cm high) were cut around the tube in six positions (Fig. 2b), i.e., a total of 36 holes. The holes in the tube had a total area of 2250 cm^2^. Half of the material cut out for the holes was not removed but instead bent to the interior of the tube at a 90° angle forming the baffles.

The tube was closed with lids on both ends (Fig. 2b), which could be dismantled when filling and emptying. A flange was glued to the tube. The flanges had diameters of 15 cm and a thickness of 1 cm. The lids had the same diameter and thickness as the flanges, and the lids and flanges were assembled and sealed with screws and bolts in plastic (Fig. 2b).

A Plexiglas rod with a diameter of 2.5 cm was placed in the center of the reactor’s whole length to stabilize it, and at the same time, it was the axis around which the reactor was turning. The rod was longer than the tube, sticking about 3 and 10 cm out through holes in the lids (Fig. 2a, d). The rod was kept in place in the middle of the tube in an open holder (Fig. 2c) fitting inside the tube. The rod was hanging in holders fixed at the container’s ends. The long end of the rod was connected to the step motor via a timing belt (Fig. 2d), and the motor driving the turn of the tube was programmed such different schemes for durations of on:off periods could be chosen.

The cation exchange membrane surrounding the tube was from Ionics. It was kept in place physically by plastic straps in the five places between the holes and at each end. The overlapping ends of the membrane sheet over the long end of the tube were sealed with silicone. The sealing was physically held in place by a 4 cm wide plate in the whole length of the reactor placed under the straps. The holes in the supporting tube (Fig. 2b) constitute the major part of the active membrane area; however, the membrane was not physically fixed to the ribbons in the support (except with the places with straps), and SSA suspension was also seen between ribbons and membrane during the experiments. Thus, the membrane area outside ribbons has added a minor part of the active membrane area; however, it was minor since the ribbons constituted a barrier to the electric field, and the active membrane area is considered as the total area of the 36 holes. The anode was a 74 cm long and 3.2 cm wide net of titanium coated with MMO (generally used for cathodic protection purposes). The strings in the metal net were 1.4 mm, and holes of the mesh were hexagonal with an area of 18 mm^2^. The anode was fastened to the internal support at the bottom of the tube. The oxygen from electrolysis at the anode inside the reactor was let out via tubes through the membrane (Fig. 2a).

The tube was placed in a container; internal size 114 cm long, 31 cm wide, and 29 cm high (when filled, the catholyte reached 23 cm height). The cathode was titanium-coated MMO rod (110 cm long and 0.3 cm in diameter) with a surface area of 104 cm^2^. It was placed parallel to the tube and close to one of the container’s lower corners (about 3 cm from the bottom and side). The distance to the center of the tube was 16 cm, i.e., the distance between the anode and cathode was between about 11 and 21 cm dependent on the position of the anode during the turning of the tube. The whole setup was placed in a fume cupboard for safety reasons to ensure not to mix and concentrate the produced O_2_ and H_2_ gases.

In summary, the reactor design differs in several parameters compared to the laboratory cells. For the dimensions, the relations between reactor and cell are (I) volume of suspension 17 (8700 ml vs. 400 ml), (II) catholyte volume 140 (70 l vs. 0.5 l), (III) active membrane area 45 (2250 cm^2^ vs. 50.2 cm^2^), and (IV) surface area of anode 74 (350 cm^2^ vs. 4.7 cm^2^). The membrane area related to the volume of the SSA suspension was doubled from 0.13 cm^2^/ml in the laboratory cells to 0.26 cm^2^/ml in the reactor. The volume of the catholyte related to the volume of SSA suspension increased from 1.25 to 31. The surface area of the anode was four times higher in the reactor per volume of SSA suspension 0.01 cm^2^/ml vs. 0.04 cm^2^/ml.

### 2C-ED experiments

Five 2C-ED experiments were performed; see Table 1.

**Table 1:** Overview of conducted ED experiments

Exp	SSA mass (kg)	Dispersion solution (l)	Liquid:solid (l/kg)	Current (A)	The turning on:off (min:min)	Duration (d)	Charge transfer (10^3^ C)	Charge transfer per mass SSA (10^3^ C/kg)
A	1.5	7.0	4.7	1.0	5:10	4	3456	230
B	1.5	7.0	4.7	3.0	5:10	4	1037	691
C	2.0	6.0	3	3.0	5:10	4	1037	518
D	3.0	5.4	1.8	3.0 (1 d) 4.5 A (3 d)	5:5	4	1425	475
E	2.0	6.0	3	3.0 (1 d) 4.5 A (2 d)	5:10	3	1037	518

The experiments varied in the mass of SSA (and since the volume of SSA suspension was constant, this means varying L:S ratio as well), the applied current, duration, and scheme of turning (on:off durations). The levels of L:S ratio were chosen based on results from (Ottosen et al. 2023) obtained in laboratory cells with 25–50 g SSA and L:S ratios of 7–14. Here, it was shown that better use of the applied current was obtained at the lowest L:S ratio (expressed as a percentage of P extracted per charge transfer), and thus, the experiments in Table 1 were chosen to have lower L:S ratios. No blind tests were made in the tube without applied current as it is known from Ottosen et al. (2023) that less than 15% P was extracted during 7 days. The current and duration were chosen to have charge transfer per mass of SSA (Table 1) similar to the previous experiments in Ottosen et al. (2023); see the discussion in paragraph 3.3.

The SSA was suspended in tap water inside the reactor. The catholyte was used for multiple experiments before being changed. The catholyte in experiment A was 70 l of 0.01 M NaNO_3_ with pH adjusted to 2 with HNO_3_. During the experiments, pH was kept below 2 in the catholyte by manually adding HNO_3_ once a day. The same catholyte remained in the plant for the next two experiments (B and C). Before starting each of the experiments, the volume of the catholyte was adjusted to 70 l by adding new tap water (during each experiment, about 2.5 L was lost due to evaporation).

At the end of the experiments, the SSA was dried and weighed. The pH and concentrations of P, Cd, Cu, Pb, and Zn were measured in the SSA. A sample was taken from the dispersion solution, and the same elements were measured in this sample.

### Characteristics of the SSA

The SSA was from the mono-incineration plant at Avedøre Spildevandscenter, a wastewater treatment plant in Copenhagen, Denmark. Here, sewage sludge is incinerated in a fluidized bed combustor at about 850 °C operated by Biofos. In the investigation, two batches (20 l buckets of SSA) were used. They were sampled on the same day and from the same place. Experiments A–C were made with SSA from batch A and experiments D and E with SSA from batch B.

The P, Cd, Cu, Pb, and Zn concentrations in the SSA were measured according to the US EPA 3015A method (U.S. EPA. 2007), and the ICP-OES used was a Varian 720-ES. The pH and conductivity of the SSA were measured by suspending 10.0 g ash in 25 ml distilled water. After 1 h of agitation and a few minutes of setting, pH and conductivity were measured directly in the suspension with Radiometer electrodes. The grain size distribution of the SSA was found by sieving (sieves: 4.0 mm, 2.0 mm, 1.0 mm, 0.5 mm, 0.25 mm, 0.125 mm, 0.063 mm, and < 0.063 mm). The particle density of the grains (> 63 µm) was measured using EN ISO 17892–3 (2015).

## Results and discussion

### Experimental ashes

Table 2 shows selected characteristics for the two batches of SSA. The pH, conductivity, and density of the two batches were very similar, and though the concentrations of the investigated chemical elements were in the same range, there were variations in the concentrations between the two batches.

**Table 2:** Characteristics of the two batches of the investigated SSA

SSA batch	pH	Conductivity (mS/cm)	Particle density (g/cm^3^)	P (g/kg)	Cd (mg/kg)	Cu (mg/kg)	Pb (mg/kg)	Zn (mg/kg)
A	9.9	2.8	2.8	58.2 ± 12.7	3.8 ± 0.1	566 ± 10	140 ± 1.2	2250 ± 79
B	9.9	2.9	2.8	54.5 ± 6.6	4.8 ± 0.6	521 ± 7	224 ± 30	2180 ± 22

The particle density of the investigated SSA of 2.8 g/cm^2^ was in the same range as the average of other SSAs found in the literature of 2.6 g/cm^2^ (Dhir et al. 2017). From the sieve analysis, it was found that 24% (SSA-A) and 11% (SSA-B) were in the fraction < 63 µm. Neither of the ashes contained particles larger than 1 mm. SSA-B was the coarsest since 12% was in the fraction 250–1000 µm, whereas it was only 3% for SSA-A. A literature survey (Dhir et al. 2017) reported that SSA consists predominantly of silt (2.5–62.5 μm) and fine sand (62.5–250 μm) size fractions, and the investigated SSA batches are thus in accordance with this. The mean concentrations in SSAs reported in the literature were 76 g P/kg, 7.7 mg Cd/kg, 1260 mg Cu/kg, 300 mg Pb/kg, and 2900 mg Zn/kg (Ma and Rosen 2021); thus, both the P concentration and the concentrations of heavy metals in the investigated ashes (Table 2) are lower than the mean values. The concentrations are well above the minimum concentrations reported from literature in Ma and Rosen (2021), meaning that the concentrations of the investigated chemical elements are in the same range as reported in the literature for SSA from other plants.

Even though the two batches of SSA were sampled in the same sampling campaign, they differed in some characteristics. The coarser SSA-B had higher concentration averages of Cd and Pb (Table 2) and the relation between the concentrations in SSA-A/SSA-B were 0.8 (Cd) and 0.6 (Pb). For comparison, the average concentrations of P, Cu, and Zn were highest in SSA-A with the relation between the two SSAs of 1.07 (P), 1.09 (Cu), and 1.03 (Zn). The operating temperature during the incineration plays the governing role in the heavy metal redistribution and stabilization from sludge to ash fractions (Udayanga et al. 2018). In Fraissler et al. (2009), the heavy metals in sewage sludge were classified into “easily volatile” (Cd and Pb) and “semi-volatile” (Cu and Zn), which are the same groupings found between the two investigated SSAs. Generally, heavy metals with lower boiling points such as Cd and Pb are distributed more in the fine fly ash particles, while most refractory heavy metals such as Cu are effectively captured from bottom or coarse fly ashes as a result of entrainment with flue gas stream rather than volatilization (Udayanga et al. 2018). The easily volatile elements could be expected in the finest ash particles, but SSA contain agglomerated, porous particles incorporating fine ash particles in the structure (Ottosen et al. 2013). Thus, a relation between heavy metal content and grainsize cannot be expected. The differences in the heavy metal concentrations suggest that SSA-B contains more volatilized and nucleated particles than SSA-A.

### SSA after treatment and extracted percentages

The concentrations of P and the investigated heavy metals in the 2C-ED treated ashes are shown in Table 3. The concentrations of P, Cd, and Cu were decreased compared to initially (Table 2). The Pb concentration, on the contrary, increased in every experiment, whereas the Zn concentration was almost unchanged in experiments B and C and decreased in the other three experiments.

**Table 3:** Mass, percentage dissolved, pH, and concentrations of the selected chemical elements in the SSA after the experiments

	Mass (kg)	Dissolved (%)	pH	P (g/kg)	Cd (mg/kg)	Cu (mg/kg)	Pb (mg/kg)	Zn (mg/kg)
A	1.25	17	3.8	53.8 ± 2.7	1.8 ± 0.2	487 ± 13	176 ± 4.6	1880 ± 104
B	0.92	39	4.5	10.6 ± 7.5	1.7 ± 0.1	438 ± 11	239 ± 6.5	2260 ± 75
C	1.4	30	4.0	21.5 ± 1.9	2.0 ± 0.1	455 ± 25	206 ± 1.0	2210 ± 147
D	2.3	24	3.5	27.3 ± 3.0	2.8 ± 0.1	460 ± 7	230 ± 5	1680 ± 17
E	1.5	25	3.7	18.5 ± 3.0	2.9 ± 0.1	440 ± 24	265 ± 18	1720 ± 140

**Fig. 3:**
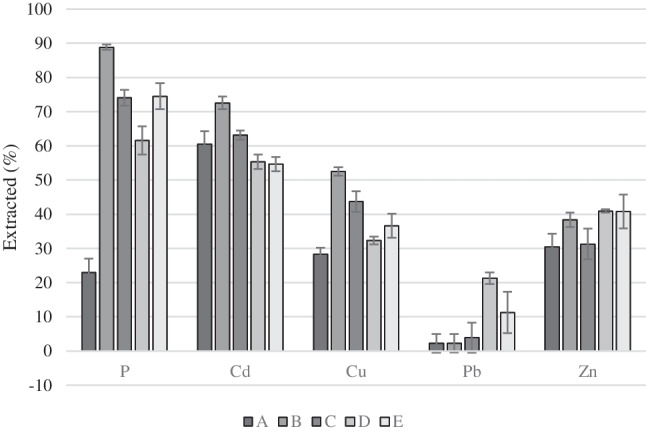
The percentwise extraction of P and heavy metals during the ED experiments. Average values and standard deviations calculated on the basis of total concentrations in the SSA after 2C-ED

Figure 3 shows the percentwise extractions calculated based on the mass of the element (concentration times the mass of SSA) in the SSA before (Table 2) and after the experiments (Table 3). The percentwise extraction is given as ((*m*_0_ − *m*_*f*_)/*m*_0_)*100%, where *m*_0_ is the initial mass of the element and *m*_*f*_ is the mass in the SSA after the experiment.

### Heavy metal extraction

The literature does not provide detailed information on the chemical speciation of heavy metals in SSAs. Thus, the different patterns for the heavy metals in Table 3 and Fig. 3 cannot be described based on the specific specifications but on more generic knowledge.

The two heavy metals with the lowest boiling point (Cd and Pb) show very different extraction patterns (Fig. 3) since Cd was extracted to the highest percentage and Pb to the lowest, i.e., there was no link between the state of the heavy metal during the ash formation and the extractability from the SSA by 2C-ED. Fly ash from fluidized bed combustion of wood has two distinctly different modes (Lind et al. 2000): a fine fly ash formed by nucleation of volatilized species (mainly KCl and K_2_SO_4_) and a coarse fly ash with irregular agglomerated particles formed from the non-volatile ash species by coalescence and agglomeration inside the char particles and on their surfaces. The coarser ash fraction of the wood fly ash (> 1 µm) was dominated by components containing non-volatile elements such as Ca, Si, Mg, Al, Fe, P, and Mn, and the agglomerate structure of the coarse ash was effective in capturing volatile species in coarse particles (Lind et al. 2000). The ash formation during fluidized bed incineration of sewage sludge may have similarities to that of wood. In Kappel et al. (2018), where SSA from the same plant as in the current investigation was used, it was reported that the SSA had a high S content (19 wt% SO_3_ measured with XRF). The Cl content was on the other hand low (0.02%). A part of the nucleated ash particles in SSA can be sulfates (as reported for the wood fly ash), and if so, the volatilized Pb and Cd could be nucleated as sulfates (since these are “easily volatile” (Fraissler et al. 2009)). In general, Cd sulfates are soluble and Pb sulfates are poorly soluble in water and acid, which can be one explanation for the difference in Cu and Pb extractions seen in Fig. 3. The low percentage of Pb extracted found during ED can also be due to the continuous precipitation of dissolved Pb with free anions in the ash suspension during the ED treatment. There are different possibilities for Pb precipitates with low solubilities, e.g., sulfates, carbonates, and phosphates (Li et al. 2021).

Cu and Zn can as “semi-volatile” be expected found both in the nucleated SSA particles and incorporated in the coarser fly ash particles. Cu and Zn sulfates are soluble in water, and if such compounds are formed, they will be extracted. It is seen that Zn had a limit of extractability of around 40% in the 2C-ED experiments, and the insoluble part may be incorporated in the coarser ash particles; however, this needs more research to be confirmed.

The extracted percentages of Cd (61–72%) and Zn (30–41%) did not vary much between the experiments compared to the other elements (Fig. 3). The Cu extraction varied between 28 and 52%, and with the same order between the experiments as P. For Pb, there was a relatively large difference between the experiments conducted with SSA1 (A, B, and C) and SSA2 (D and E) in that the extraction was very low (< 4%) from SSA1 and higher (21 and 11%) from SSA2. Thus, the higher Pb content in SSA-B had a higher percentage of extractable Pb. The differences between the heavy metal extractions underline the differences in speciations in SSA.

### Charge transfer and extracted P

The P extraction ranged from 23 (experiment A) to 89% (experiment B). The target of 80% P extraction in the dispersion solution was thus obtained in experiment B if the loss to the catholyte was less than 9%. It seems likely when compared to the laboratory results in Ottosen et al. (2016) (with SSA from the same plant and a loss of less than 9% P to the catholyte at a similar extraction percentage).

The extracted percentage of P is higher than that of the heavy metals except from experiment A (with the lowest charge transfer per mass of SSA, Table 2). In this experiment Cd, Cu, and Zn were extracted in higher percentages than P, showing that part of these heavy metals was present in soluble particles (e.g., as sulfates as discussed), which are not as dependent on the acidification as the solubilization of e.g. the calcium phosphates (reported present in many SSAs).

**Fig. 4:**
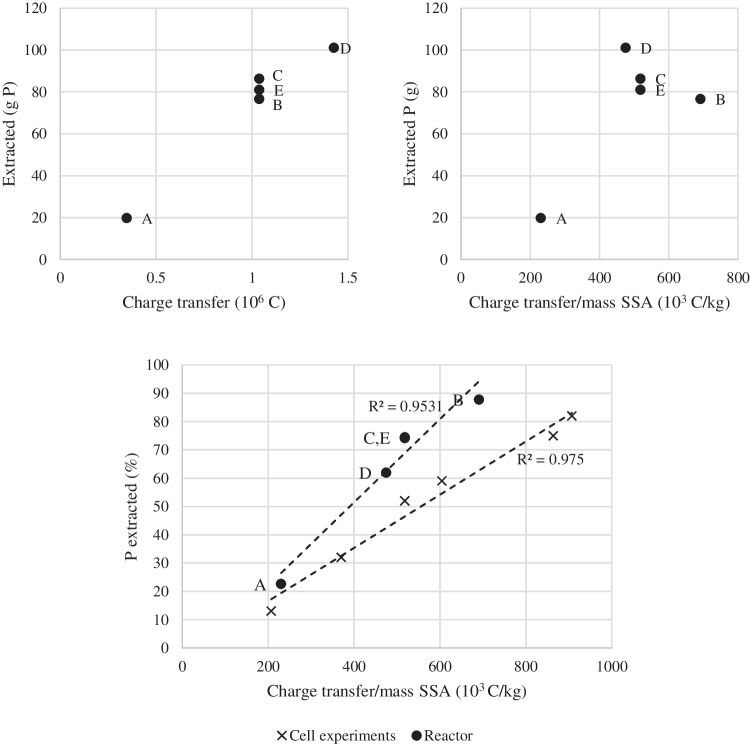
**a** Extracted mass of P vs. charge transfer, **b** extracted mass of P vs. charge transfer per unit mass of SSA, and **c** extracted percentage of P vs. charge transfer per unit mass of SSA for experiments A–E and 2C-ED cell experiments from [22]

In [22], it was reported that the current and duration were the two process variables with the most influence on the P extraction result during 2C-ED of SSA. The charge transfer is the current time duration and is thus calculated from the two major variables for the process. The extracted mass of P in each experiment related to the charge transfer is shown in Fig. 4a. The general trend is, as expected, the higher the charge transfer, the more P is extracted. It is also seen that the extracted mass of P differed between experiments B, C, and E even though the same charge transfer was applied. From Table 1, it can be seen that these three experiments varied in the mass of SSA treated (and thus L:S) and the current applied. Figure 4b shows the extracted mass of P related to the charge transfer per mass of SSA. The extracted P mass was lowest in experiments A and B. Since these two experiments were carried out with the highest L:S ratio of 4.7 (Table 1), it underlines that better use of the current related to P extraction is obtained when treating a thicker SSA suspension (lower L:S levels) in the reactor as well. This is consistent with Ottosen and Lima (2021) and Ottosen et al. (2023), where the roles of the H^+^ ions explain this finding. They can be used for extraction (ash dissolution) or be current carrying from the suspension towards the anode (and thus lost in terms of the P extraction). In thin suspensions, a higher fraction of the current carrying H^+^ ions must be expected than in thicker suspensions (at the same applied current), and thus, the extraction related to charge transfer is most efficient in the thicker suspensions.

Figure 4c shows the percentage of extracted P versus the charge transfer per mass unit of SSA for experiments A to E in the reactor, together with results from 2C-ED experiments in the small laboratory cell from [22] treating 25 to 50 g SSA (experiments no. 6, 9, 15, 17, and 18). It is seen that the relation is linear for both experimental series with *R*^2^ at 0.9531 and 0.9750, respectively. The SSA in [22] was from the same plant as the SSA of the current paper, but the P concentration was higher in that batch 82.8 g P/kg compared to the 54.5 and 58.2 mg P/kg in the SSAs of the current investigation. This needs to be taken into account when comparing the result in Fig. 4c; however, the line is steepest for the extraction in the reactor, which indicates a more effective use of the applied current. As discussed, treating a suspension with a low L:S ratio is preferable, and the L:S ratio was lowest in the tube (1.8 to 4.7, Table 1) compared to the lab (7 to 14 [22]). This is expected to have a major influence related to the better utilization of the charge transfer in the reactor (Fig. 4c). In Fig. 4c, the highest SSA mass per membrane area in the tube reactor and laboratory cell was 1.3 g/cm^2^ and 1.0 g/cm^2^, respectively (3000 g/2250 cm^2^ vs. 50 g/50.2 cm^2^), i.e., in the same range. This supports that it is the L:S ratio rather than the mass treated that is most important to the result.

The more efficient use of the applied current may additionally be due to the different designs between cell and reactor. Two major differences influencing the process to the benefit of the reactor are expected. (I) The larger surface area of the anode per volume SSA in the reactor: the anode mesh in the tube reactor likely distributed the electric field better in the SSA suspension than the bar electrode in the laboratory cell, both due to the larger surface area per volume SSA suspension and the placement in the tube parallel to the CEM and cathode throughout the length. (II) The larger surface area of the membrane combined with the larger catholyte volume per volume of SSA suspension: the CEM is known to be of major importance to the overall potential difference between the electrodes in 2C-ED (Ottosen and Lima 2021), especially together with the fluctuations in catholyte pH (increases from electrolysis and daily adjustments to pH 2). The variation in potential difference due to these pH fluctuations suggests fouling/precipitation at the cathode side of the CEM when pH increases to neutral and alkaline levels (Ottosen and Lima 2021), and the fouling may influence the separation process; however, it remains to be documented. The larger membrane area and catholyte volume in the reactor relative to the volume of the treated SSA suspension must be expected to have decreased the fouling/precipitation.

### Heavy metal content in the dispersion solution

**Fig. 5:**
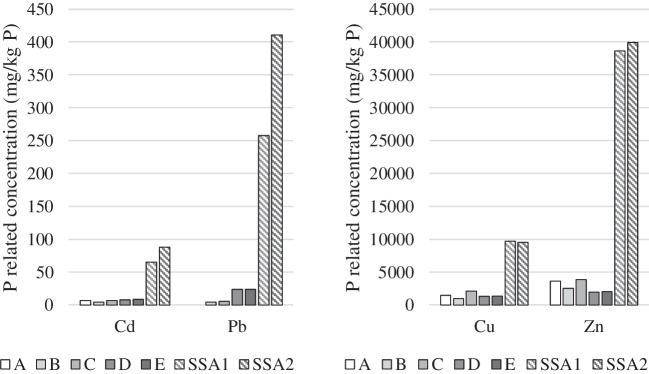
The concentration of heavy metals relative to P in the dispersion solution after the 2C-ED experiments and in the two SSA batches before treatment

The heavy metal concentration relative to the P concentration is important when a fertilizer product is made from waste in order not to add contamination to agricultural land. The Danish Ministry of Environment has limiting values for Cd and Pb concentrations related to the P concentration for use of waste on agricultural land: 100 mg Cd/kg P and 10 g Pb/kg P (Danish Ministry of Environment 2018). Figure 5 shows the concentration of Cd, Cu, Pb, and Zn relative to P for the samples taken from the dispersion solution in the 2C-ED reactor at the end of the experiments and the similar relation in the two SSAs before the ED experiments. It is seen that the limiting values were already met in the two investigated SSA batches. It is also seen that the P-related concentration decreased significantly from SSA to dispersion solution for all four heavy metals. This decrease relates to the simultaneous removal of the extracted heavy metals into the catholyte, but also and especially for Pb because a lower fraction of the heavy metals than P was extracted (Fig. 3).

## Conclusions

A new setup based on 2C-ED was developed and tested for the simultaneous extraction of P and separation of heavy metals from SSA by electrodialysis. The setup was designed for the treatment of an SSA suspension with a low liquid:solid ratio (the lowest tested here was 1.8). The new design was based on placing the suspension in a rotating reactor to keep the SSA suspended. The reactor design can be developed into a continuous process when upscaled. Up to 89% P was extracted. The results indicated that the applied DC current was used more efficiently than in the previous smaller laboratory cell experiments (up to 50 g SSA). A good separation of heavy metals was obtained. The final solution with the extracted P had concentrations of heavy metals, which by far met the limiting values for spreading on agricultural land. With these good results, the reactor design can be a base for the upscaling of the 2C-ED process to pilot scale.

## Data Availability

The authors confirm that the data supporting the findings of this study are available within the article.
